# Current strategies in the therapeutic approach for adenocarcinoma of the ampulla of Vater

**Published:** 2013-09-25

**Authors:** B Gaspar, M Beuran, S Paun, R Ganescu, S Hostiuc, I Negoi

**Affiliations:** * “Carol Davila" University of Medicine and Pharmacy Bucharest Emergency Hospital of Bucharest Romania; ** National Institute of Legal Medicine

**Keywords:** ampulla of Vater, cancer, therapy

## Abstract

**Introduction:** Ampulla of Vater tumors, neoplastic diseases located at the confluence of the common bile duct with the main pancreatic duct; represent 0.2% of all gastrointestinal cancers.

**Method: **Retrospective study of all patients admitted in the Emergency Hospital of Bucharest Romania between January 2008 and January 2013, the only selection criterion used being a pathology report which describes an ampulla of Vater carcinoma. We have also performed a review of the medical literature up to 2013, using the PubMed/Medline, Proquest Hospital Collection, Science Direct, Cochrane Library and Web of Science databases. We have used different combinations of the following keywords: “ampulla of Vater", “carcinoma", “resection", reviewing the reference list of retrieved articles for further relevant studies.

**Results:** Forty eight patients with ampulla of Vater carcinoma were identified, of whom 59.6% men, 71% from urban areas, and a mean age of 66 ± 13.3 years. Most patients were admitted for obstructive jaundice (49%), right upper quadrant abdominal pain (19%), nausea and loss of appetite in 13%, loss of weight (13%) and upper digestive obstruction in 6% of cases. All patients were evaluated with abdominal transparietal ultrasonography and double contrast, pancreatic protocol, Mutidetector Row Computed Tomography. The abdominal Magnetic Resonance Imaging was performed in 10 cases, upper gastrointestinal endoscopy in 9 cases, and Endoscopic Retrograde Cholangiopancreatography in 39 cases. According to the AJCC Cancer Staging 9% were into stage I, 47% into stage II, 40% into stage III and 4% into stage IV of the disease. The therapeutic approach was surgical for 44 patients and an endoscopic palliation with stent insertion in 4 cases. The surgical procedure was represented by Whipple pancreatoduodenectomy in 27 cases, pylorus preserving pancreatoduodenectomy in 15 cases and exploratory laparotomy in 2 cases. Early morbidity was represented by pancreatic leakage in 4 cases.

**Conclusions:** There are clinical scenarios in which it is quite challenging to distinguish a primary ampullary adenocarcinoma based on a preoperative workup. Nevertheless, an aggressive approach should be performed, knowing the higher resectability rates and a five-year survival for these patients. Complete surgical resection should be performed in all medically fit patients, candidates for pancreatoduodenectomy, by a high volume, trained surgeon, able to offer a low morbidity and mortality.

## Introduction

Periampullary tumors are neoplastic diseases, benign and malign, arising around the confluence of the common bile duct with the main pancreatic duct, named the ampulla of Vater. The periampullary tumors may have a different origin, at the level of the pancreatic head in 60%, ampulla of Vater in 20%, distal common bile duct in 10% and duodenum in 10% of the resected specimens. But this distribution is relative, because tumors of the duodenum, ampulla of Vater and distal common bile ducts have a higher frequency of resectability compared to the pancreatic head cancers, which represent as much as 90% from the resectable and unresectable periampullary neoplasia [**1,2**]. 

 The clinical picture is dominated by an early occurrence of jaundice, contributing to the early detection and a higher resection rate. Any jaundiced patient over 40 years old should be carefully investigated to preclude a malignancy, and the clinician should remember that as much as 25% of patients with periampullary neoplasm have the onset of disease with an episode of acute pancreatitis [**3**]. In patients with familial adenomatous polyposis, the papilla of Vater carcinoma is the main location for malignant occurrence after proctocolectomy [**4**]. 

 The adenocarcinoma of the ampulla of Vater seems to follow the same adenoma–carcinoma sequence, residual adenomatous areas being found in up to 91% of ampullary carcinoma and conversely, 50% of benign ampullary neoplasia harboring a malignant component [**5**]. Henson et al. analyzed whether carcinomas of the pancreas, gallbladder, extrahepatic bile ducts, and ampulla of Vater share a field for carcinogenesis [**6**], identifying an incidence of 11.71, 1.43, 0.88 and 0.49 per 100000 persons at risk for pancreatic, gallbladder, extrahepatic bile ducts and ampullary carcinomas, respectively. The authors conclude that these cancers have a common embryonic cellular ancestry, differentiation pathways, mucosal histologic patterns and population-related tumor development indicating a field effect in carcinogenesis. The rate of cancer development is similar in all four sites, even though the absolute incidence rate varies. Irrespective of the location, the ductal epithelium has the same malignant transformation rate,, the only difference being determined by the relative surface area of the mucosa. Therefore, in the periampullary region pancreatic head cancers are prevalent due to pancreatic ductal mucosa which has a greater surface area [**6**]. 

 The macroscopic appearance of the ampullary carcinomas may be: (a) intramural tumors – inside the ampulla, without any protrusion inside the duodenum, (b) extramural tumors – polypoid tumors protruding through the ampullary orifice into the duodenum, (c) ulcerative cancers of the ampulla [**7**]. Kayahara et al. found a significant relationship between the gross appearance of the tumor and nodal involvement: 22% lymphatic invasion for protruding type, 42% for mixed type and 100% for ulcerative type [**8**]. The 5-years survival rate also correlates with the macroscopic appearance of the tumor: 75% for protruding type, 49% for mixed type and 17% for ulcerative carcinomas [**8**]. 

 According to the microscopic classification, there are two main histologic types of carcinoma of the papilla of Vater: (a) intestinal type – similar to tubular carcinoma of the stomach or the colon, (b) pancreatobiliary type – characterized by papillary projections with scant fibrous cores [**9**]. For the differentiation between these two types is also useful the immunohistochemical staining for apomucin of the mucosa (MUC2) and keratin types (CK7 and CK20) [**10**]. 

 Fischer and Zhou presented an immunohistochemical marker profile for intestinal type of keratin 7-, keratin 20+, MUC2+ and for pancreaticolbiliary type of kertin7+, keratin 20- and MUC2- [**4**]. According to de Paiva Haddad et al. the most accurate markers are CDX2, MUC1 and MUC2 [**11**]. They have observed a higher expression for MUC2 (74.4% versus 23.4%), CK20 (76.7% versus 29.8%), CDX2 (86% versus 21.3%), and CD10 (81.4% versus 51.1%) in intestinal type cancers and a higher expression for MUC1 (53.5% versus 82.9%) and CK7 (79.1% versus 95.7%) in pancreaticobiliary adenocarcinomas [**11**]. 

 The different published series presents a variable distribution of 49% and 21%, 25% and 75%, 26.9% and 44.8% for the intestinal type and the pancreaticobiliary type, respectively [**4,9**]. 

 Abdominal Computed Tomography, strictly adhering to the pancreatic protocol, is very useful for diagnosis and pretherapeutic staging of the periampullary tumors. This have to be performed with oral and intravenous contrast material, during one breath-hold, with 1-3 mm slices performed during arterial and venous contrast enhancement [**12**]. 

 Magnetic retrograde colangiopancreatography may offer additional details about papilla of Vater and surrounding structures [**13**]. Sugita et al. found that Magnetic Resonance (MR) images clearly characterize the normal structures of the ampullary region, including the Oddi muscle, duodenal wall, common bile duct and pancreas [**14**]. Sensitivity, specificity, accuracy, positive predictive value, and negative predictive value of high-spatial resolution MR imaging for the evaluation of the tumor invasion into the surrounding structures were 88%, 100%, 96%, 100%, and 94%, respectively [**14**]. 

 Transabdominal ultrasonography has a lower sensitivity; for example, a study conducted by Qiao et al.l found the ampullary mass only in 10 out of 127 patients [**15**]. Skordilis et al. found conventional ultrasound to have a detection rate for ampullary cancer of only 15% compared with a rate of 100% for endoscopic ultrasound [**16**]. 

 Endoscopic cholangiopancreatography is very useful for the examination, for taking biopsies, performing cholangiopancreatography and for stent insertion when appropriate, but the clinician should never forget its false negative rate for biopsies, as high as 50% [**17-19**]. 

 Nowadays, studies investigating the applicability of natural orifice transluminal endoscopic peritoneoscopy for the staging of pancreatic head masses are under investigation, finding a high concordance with laparoscopic exploration for the assessment of peritoneal metastases [**20**].

## Method

For this retrospective study, we have selected all patients admitted in the Emergency Hospital of Bucharest Romania, between January 2008 and January 2013, with a pathology report describing an ampulla of Vater carcinoma, even if the specimen was obtained after a surgical resection or after an endoscopic biopsy. We have excluded all the patients with a pancreatic protocol Computed Tomography suggesting another type of periampullary tumor and secondary invasion of the ampulla of Vater. We have reviewed all the patients’ data file, registering all the demographic data, clinical picture, diagnostic workup, therapeutic approach, early and late morbidity of the surgical versus endoscopic treatment, 30 days mortality. The patients were considered part of the surgical group if the primary therapeutic approach was a laparotomy, with a palliative (biliary, digestive or double by-pass for locally advanced or metastatic disease) or radical procedure (pancreatoduodenectomy). Patients were considered part of the endoscopic group if the primary procedure was the insertion of a biliary stent for the biliary decompression. Patients with cholangitis and stent insertion were considered from the surgical group if after stent insertion a laparotomy was performed. Patients without cholangitis with a stent insertion and a secondary laparotomy were considered a failure of the endoscopic treatment.

 Statistical analysis was performed with the statistical analysis program IBM SPSS Statistics, 20.0. Continuous variables are presented as mean ± standard deviation, and the categorical variables as number (percentage). For normal distributed data we have used independent samples T-test or ANOVA tests and for nonparametric data Mann-Whitney U or Kruskal-Wallis H tests. The categorical variables were analyzed by Chi-square or Fisher’s exact test, where appropriate. The probability p to decline the null hypothesis was set at 0.05.

## Results

**Fig. 1:**
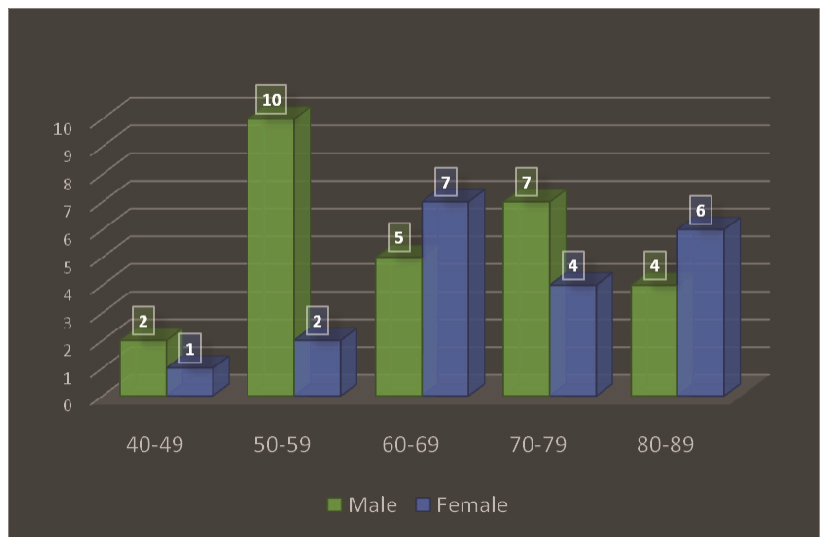
Age distribution groups

 During 5 years we have selected 48 patients cu ampulla of Vater carcinoma, 59.6% (29 patients) men, 71% (34 patients) from the urban area, with the mean age of 66 ± 13.3 years old (**Fig. 1**).

 Most of the patients were admitted for obstructive jaundice (49%), right upper quadrant abdominal pain (19%), nausea and loss of appetite in 13%, loss of weight (13%) and upper digestive obstruction in 6% of the cases. We may observe a lower percentage of patients admitted for jaundice in our study, compared to the study of Monson et al. who presented a frequency of 67% [**21**].

 The clinical exam revealed a jaundiced patient in 39% of the patients, an abdominal tumor in 4.2%, 8.3% the Courvoisier-Terrrier sign, 19% abdominal pain on palpation and was nonspecific in 43%.

**Fig. 2:**
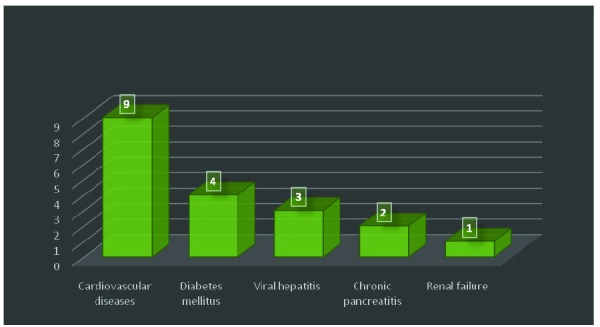
Preexisting morbidities of the patients

 33% of the patients were smokers. The preexisting morbidity of the patients was represented by chronic pancreatitis in 2 cases, chronic viral hepatitis in 3 cases, diabetes mellitus in 4 cases, renal failure in 1 case and cardiovascular diseases in 9 cases (**Fig. 2**).

**Fig. 3:**
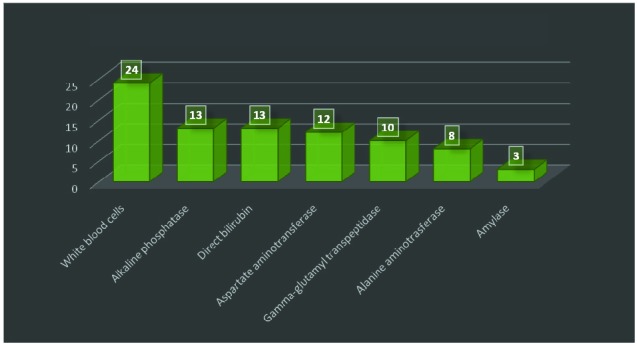
Blood samples on admission

 Blood samples on admission revealed high values for: white blood cells (24 patients), alkaline phosphatase (13 patients), direct bilirubin (13 patients), aspartate aminotransferase (12), alanine aminotransferase (8 patients), gamma-glutamyl transpeptidase (10 patients), amylase (3 patients) (**Fig. 3**).

**Fig. 4:**
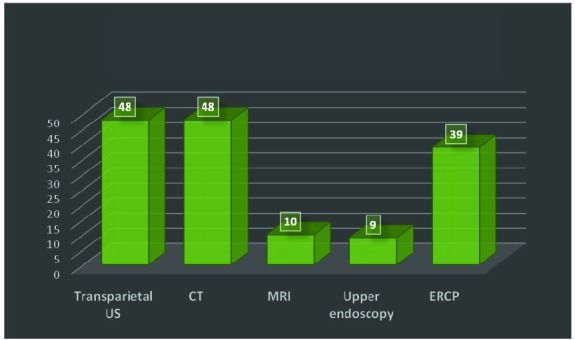
Diagnostic & staging imagistics

 During positive diagnosis and staging workup all patients were evaluated with abdominal transparietal ultrasonography and double contrast, pancreatic protocol, Mutidetector Row Computed Tomography. Abdominal ultrasonography on admission revealed liver metastases in only 45 of cases. Abdominal Magnetic Resonance Imaging was performed in 10 cases, upper gastrointestinal endoscopy in 9 cases, and Endoscopic Retrograde Cholangiopancreatography in 39 cases (**Fig. 4**).

**Fig. 5:**
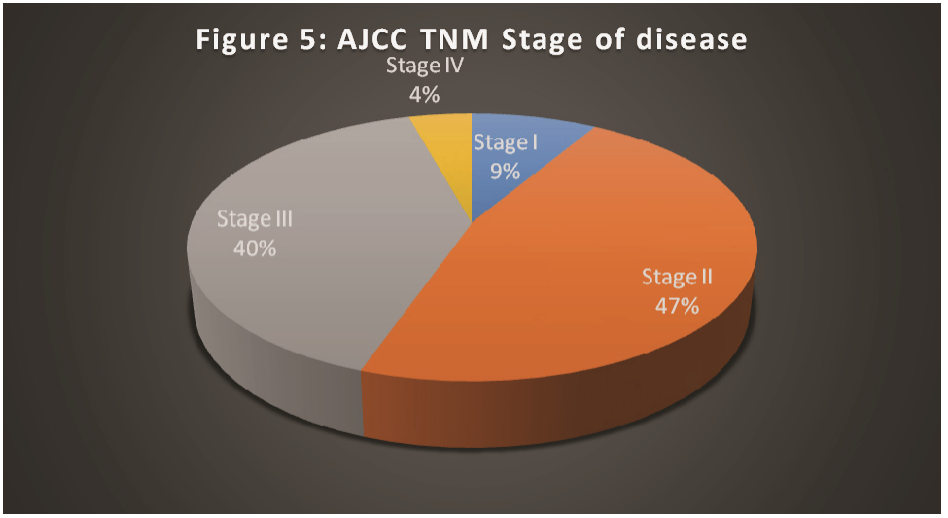
AJCC TNM Stage of disease

 According to the AJCC Cancer Staging Manual 7th Edition, 9% (4 patients) were in stage I, 47% (22 patients) in stage II, 40% (19 patients) in stage III and 4% (2 patients) in stage IV of the disease (**Fig. 5**) [**22**].

**Fig. 6:**
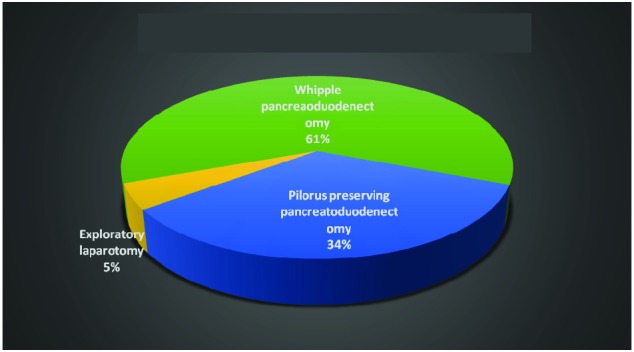
Surgical group

 The therapeutic approach for the study group was surgical for 44 patients and endoscopic palliation with stent insertion in 4 cases. The surgical procedure was represented by Whipple pancreatoduodenectomy in 27 cases, pylorus preserving pancreatoduodenectomy in 15 cases and exploratory laparotomy in 2 cases (**Fig. 6**). 

 Early morbidity was represented by pancreatic leakage in 4 cases.

## Discussion

There are no recommendations regarding the management of ampullary cancers, neither in the National Comprehensive Cancer Network (NCCN), from the United States, nor from the European Society for Medical Oncology (ESMO) [**23**]. The resection of an ampullary tumor may be through pancreatoduodenectomy (standard approach), local resection and minimally invasive nonsurgical therapies (endoscopic snare resection, laser ablation, photodynamic therapy) [23,24]. Hospital mortality after pancreatic head resection in high volume centers was below 5% (**Table 1**) [**25-30**] and the reported postoperative morbidity ranged from 25% up to 60% (**Table 2**) [**19,31,32**].

 The local excision of the ampulla may be performed in high risk patients, unfitted for major resection. Zhong et al. presented a 3 and 5 year overall survival of 54% and 21%, and a 3 and 5 year local control of 36% and 31%, respectively [**33**].

**Table 1:** Morbidity and mortality of the pancreatoduodenectomy performed for ampulla of Vater cancer [**7,29,32,34,35**].

Study	Number of patients	Morbidity	Mortality
Yamaguchi et al.	107	-	5.8%
Talamini et al.	106	47%	3.8%
Beger et al.	98	-	3.2%
Nagakawa et al.	1423	-	1.3%
Di Giorgio et al.	94	48%	9.3%

**Table 2:** . Early morbidity of the pancreatoduodenectomy performed for ampulla of Vater cancer [**34,36,37**].

Study	Number of patients	Pancreatic leakage	Biliary leakage
Talamini et al.	106	25%	3%
Roberts et al.	32	31%	-
Farnell et al.	2	20%	20%

 Analyzing the surgical strategy based on lymphatic spread on 36 patients with papilla of Vater carcinomas Kayahara et al. showed that 42% of the patients had nodal involvement, the lymph nodes with the highest metastatic rates being the inferior pancreaticoduodenal nodes (number 13b – in 31% of patients) and the superior mesenteric lymph node (number 14 – for 17% of the patients). The 5-years survival rate was 74% in the absence of nodal involvement and 31% for nodal metastasis group [**8**].

 Studying the clinicopathological and immunohistochemical characteristics of the ampulla of Vater carcinomas, Roh et al. found a 5 year survival rate of 58.8% [**10**]. Out of 34 patients, 12 had intestinal type and 22 had pancreatobiliary type, the long-term survival after the curative resection being significantly higher for intestinal type cancers [**10**].

 De Paiva Haddad et al. assessed the best immunohistochemical panel for tumor classification and the survival differences between groups [**11**]. After the immunohistochemical staining for 97 patients, 43 were intestinal type carcinoma and 47 pancreaticobiliary. In this study, the survival was significantly worse in pancreaticobiliary type (p = 0.021), but the multivariate analysis showed that only lymph node metastasis, lymphatic invasion and stage were independent risk factors for survival [**11**].

**Table 3:** The 5 year-survival for radical resection of the ampulla of Vater cancers [32,35,38].

TNM Staging		5 year-survival	
	Beger et al.	Nagakawa et al.	De Castro et al.
Stage I	84%	75%	75%
Stage II	70%	48%	66%
Stage III	27%	34%	35%
Stage IV	0%	19%	0%

## Conclusions

There are clinical scenarios in which it is quite challenging to distinguish a primary ampullary adenocarcinoma based on preoperative workup. Nevertheless, an aggressive approach should be performed, knowing the higher resectability rates and the five-year survival for these patients. Complete surgical resection should be performed in all medically fit patients, candidates for pancreatoduodenectomy, by a high volume, trained surgeon, able to offer a low morbidity and mortality.
